# Supplementary material for: Effects of a self-guided digital mental health self-help intervention for Syrian refugees in Egypt: A pragmatic randomized controlled trial
Source: PLoS Med. 2024 Sep 9;21(9):e1004460. doi: 10.1371/journal.pmed.1004460 (PMC11419380; doi:10.1371/journal.pmed.1004460)
Supplement: S1 CONSORT Checklist — (PDF) [file pmed.1004460.s001.pdf]

Reporting checklist for a randomised trial of a social or psychological intervention.

Based on the CONSORT-SPI guidelines.

Instructions to authors

Complete this checklist by entering the page numbers from your manuscript where readers will find each of the items listed below.

Your article may not currently address all the items on the checklist. Please modify your text to include the missing information. If you are certain that an item does not apply, please write "n/a" and provide a short explanation.

Upload your completed checklist as an extra file when you submit to a journal.

In your methods section, say that you used the CONSORT-SPIreporting guidelines, and cite them as:

Montgomery P, Grant S, Mayo-Wilson E, Macdonald G, Michie S, Hopewell S, Moher D; on behalf of the CONSORT-SPI Group. Reporting randomised trials of social and psychological interventions: the CONSORT-SPI 2018 Extension. Trials. 2018;19:407.

		Reporting Item	Section and paragraph
**Title and Abstract**			
Title	#1a	Identification as a randomised trial in the title.	**Title**
Abstract	#1b	Structured summary of trial design, methods, results, and conclusions. Refer to CONSORT extension for social and psychological intervention trial abstracts (https://trialsjournal.biomedcentral.com/articles/10.1186/s13063-018-2735-z/tables/3)	**Abstract**
**Introduction**			
Background and objectives	#2a	Scientific background and explanation of rationale	**Section 1, para. 1-5**

Background and objectives	#2b	Specific objectives or hypothesis, If pre-specified, how the intervention was hypothesized to work.	**Section 1, para. 6**
**Methods**			
Trial design	#3a	Description of trial design (such as parallel, factorial) including allocation ratio. If the unit of random assignment is not the individual, please refer to CONSORT for Cluster Randomised Trials (https://www.equator-network.org/reporting-guidelines/consort-cluster/)	**Section 2.1**
Trial design	#3b	Important changes to methods after trial commencement (such as eligibility criteria), with reasons	**n/a**
Participants	#4a	Eligibility criteria for participants. When applicable, eligibility criteria for settings and those delivering the interventions	**Section 2.2**
Participants	#4b	Settings and locations of intervention delivery and where the data were collected	**Section 2.2**
Interventions	#5	The interventions for each group with sufficient details to allow replication, including how and when they were actually administered	**Section 2.4**
Outcomes	#6a	Completely defined pre-specified outcomes, including how and when they were assessed	**Section 2.3**
Outcomes	#6b	Any changes to trial outcomes after the trial commenced, with reasons	**Section 2.1**
Sample size	#7a	How sample size was determined.	**Section 2.2**
Sample size	#7b	When applicable, explanation of any interim analyses and stopping guidelines	**n/a**
Randomization - Sequence generation	#8a	Method used to generate the random allocation sequence.	**Section 2.1**
Randomization - Sequence generation	#8b	Type of randomization; details of any restriction (such as blocking and block size)	**Section 2.1**

Randomization - Allocation concealment mechanism	#9	Mechanism used to implement the random allocation sequence, describing any steps taken to conceal the sequence until interventions were assigned	**Section 2.1**
Randomization - Implementation	#10	Who generated the allocation sequence, who enrolled participants, and who assigned participants to interventions	**Section 2.1**
Awareness of assignment	#11a	Who was aware after assignment to interventions (for example, participants, providers, those assessing outcomes), and how any masking was done	**Section 2.1**
Awareness of assignment	#11b	If relevant, description of the similarity of interventions	**n/a**
Analytical methods	#12a	Statistical methods used to compare group outcomes. How missing data were handled, with details of any imputation method	**Section 2.5**
Analytical methods	#12b	Methods for additional analyses, such as subgroup analyses, adjusted analyses, and process evaluations	**Section 2.5**
Interventions	#5a	Extent to which interventions were actually delivered by providers and taken up by participants as planned	**Section 3.1**
Interventions	#5b	Where other informational materials about delivering the interventions can be accessed	**Section 1, para. 5**
Interventions	#5c	When applicable, how intervention providers were assigned to each group	**n/a**
**Results**			
Participant flow diagram (strongly recommended)	#13a	For each group, the numbers randomly assigned, receiving intended treatment, and analysed for the outcomes. Where possible, the number approached, screened, and eligible prior to random assignment, with reasons for non-enrolment	**Section 3.1, para. 2**
Participant	#13	For each group, losses and exclusions after randomization,	**Section 3.1,**

flow	b	together with reasons	**para. 1**
Recruitment	#14a	Dates defining the periods of recruitment and follow-up	**Section 3.1, para. 1**
Recruitment	#14b	Why the trial ended or was stopped	**Section 3.1, para. 1**
Baseline data	#15	A table showing baseline characteristics for each group. Include socioeconomic variables where applicable	**Section 3.1, para. 2**
Numbers analysed	#16	For each group, number included in each analysis and whether the analysis was by original assigned groups	**Section 3.1, para. 1**
Outcomes and estimation	#17a	For each outcome, results for each group, and the estimated effect size and its precision (such as 95% confidence interval). Indicate availability of trial data	**Sections 3.2 and 3.3**
Outcomes and estimation	#17b	For binary outcomes, presentation of both absolute and relative effect sizes is recommended	**n/a**
Ancillary analyses	#18	Results of any other analyses performed, including subgroup analyses and adjusted analyses, distinguishing pre-specified from exploratory	**3.4**
Harms	#19	All important harms or unintended effects in each group (For specific guidance see CONSORT for harms)	**Section 3.4, para. 2**
**Discussion**			
Limitations	#20	Trial limitations, addressing sources of potential bias, imprecision, and, if relevant, multiplicity of analyses	**Section 4, para. 5**
Interpretation	#22	Interpretation consistent with results, balancing benefits and harms, and considering other relevant evidence	**Section 4, para. 2-4**
Generalisability	#21	Generalisability (external validity, applicability) of the trial findings	**Section 4, para. 6**

Important

**information**			
Registration	#23	Registration number and name of trial registry	**Section 2.1**
Protocol	#24	Where the full trial protocol can be accessed, if available	**Supplementary 1**
Declaration of interests	#25	Sources of funding and other support, role of funders. Declaration of any other potential interests	**Section 5**
Stakeholder involvement	#26a	Any involvement of the intervention developer in the design, conduct, analysis, or reporting of the trial	**Section 5**
Stakeholder involvement	#26b	Other stakeholder involvement in trial design, conduct, or analyses	**n/a**
Stakeholder involvement	#26c	Incentives offered as part of the trial	**n/a**

The CONSORT-SPI checklist is distributed under the terms of the Creative Commons Attribution License CC-BY. This checklist was completed on 03. January 2024 using https://www.goodreports.org/, a tool made by the EQUATOR Network in collaboration with Penelope.ai
