# Supplementary material for: Effects of a self-guided digital mental health self-help intervention for Syrian refugees in Egypt: A pragmatic randomized controlled trial
Source: PLoS Med. 2024 Sep 9;21(9):e1004460. doi: 10.1371/journal.pmed.1004460 (PMC11419380; doi:10.1371/journal.pmed.1004460)
Supplement: S1 Study Protocol — (PDF) [file pmed.1004460.s002.pdf]

Title

Randomized controlled trial to test the (cost-)effectiveness of Step-by-Step, a smartphone-based self-help program for Syrian refugees.

Study protocol

1. Purpose

1.1 Summary for the public

Worldwide, there is a large disparity between the high number of people affected by psychological distress, and the small percentage of people with access to psychological support. This mental health care gap is particularly problematic among refugee populations. Easily accessible, affordable and effective psychological intervention approaches are needed. Internet- and smartphone-based programs are a promising approach to provide basic support and self-help skills to a large number of people. Previous research shows that internet-based psychological support-programs are effective in treating a variety of common psychological problems.

In the EU-funded STRENGTHS project, researchers at Freie Universität Berlin (Prof. Christine Knaevelsrud) in Germany create and test a smartphone app for Syrian refugees. The project is supported by researchers at the London School of Hygiene and Tropical Medicine (Prof. Bayard Roberts) in the UK. This app is based on Step-by-Step, a brief and low-intensity self-help programme developed by the WHO. It is available in Arabic and English for Android, iOS and Web. Users of Step-by-Step receive a series of 5 weekly sessions with illustrated narratives and interactive exercises. A team of trained Syrians ("e-helpers") is available in case of specific questions, for example regarding the program, the study or technical issues. Participants can contact e-helpers using the built-in text and voice messaging system of the app.

Before Step-by-Step can be made available widely, this study will test the effect that the use of the support-program has on participants' psychological distress and problems in daily life. Furthermore, the effect on the use of other mental health care services and the satisfaction of participants with Step-by-Step will be investigated. For this research, half of the participants will receive full access to Step-by-Step (group 1) while the other half of the participants will receive access to information about their symptoms, and on how to get help when experiencing difficult emotions or problems (group 2).

1.2 Scientific purpose / objectives

Overall objective:
The EU-funded STRENGTHS project is a joint effort by academic and research institutions from Europe, international agencies and humanitarian organizations. The overall objective of STRENGTHS is to scientifically investigate different approaches to improving the responsiveness of health care systems to the Mental Health and Psychosocial Support (MHPSS) needs of Syrian refugees. This proposed study is part of the STRENGTHS project and aims to evaluate Step-by-Step, a smartphone-based self-help program designed to support Syrian refugees with increased psychological distress and impaired functioning.

Primary objective:
The main objective is to test the effectiveness and cost-effectiveness of SbS by comparing it against a treatment-as-usual (TAU) control group.

Secondary objectives:
Furthermore, the study will describe existing MHPSS systems for Syrian refugees in Egypt and how Syrians refugees obtain MHPSS care in the country. This is done to identify barriers for seeking and receiving care as well as bottlenecks in providing Syrian refugees with mental health care. By exploring the views of participants and stakeholders through process evaluation, the study aims to identify ways of improving the program and ways of scaling up SbS in the Egyptian health care system.

Global objectives:
Parallel studies on the SbS app are being carried out in Germany, Sweden and Lebanon. In addition, partners in STRENGTHS are implementing a variety of other approaches to refugee mental health care (e.g. face-to-face group interventions) in the Netherlands, Switzerland, Turkey, Lebanon and Jordan. The global objective of STRENGTHS is to combine the results of these studies in order to generate a set of comprehensive evidence-base for approaches to refugee MHPSS in different country contexts.

1.3 Research questions

Primary research questions:
1) To which extent contributes SbS to the reduction of symptoms of psychological distress and functional impairment three months after completing the program?
2) To which extent contributes SbS to the reduction of costs of care in the twelve months after entering the study?

Secondary research questions:
1) To which extent contributes SbS to the reduction of symptoms of PTSD and self-defined problems three months after completing the program?
2) What is the moderating role of sociodemographic factors, trauma exposure and post-migration stressors in the effects of SbS?
3) How satisfied are participants with SbS, how are the different features of the app utilized and how do participants rate the usability of the program?
4) What MHPSS services are available to Syrian refugees in Egypt and what are reasons for not accessing or receiving them?
5) What are barriers and facilitators to scaling up SbS in the Egyptian health care system?

2. Methods

2.1 Description of methods, assessments, techniques or treatment
The study combines qualitative and quantitative approaches to conduct a health systems analysis as well as a randomized controlled trial (RCT) with subsequent process evaluation and additional interviews on scaling up.

Health systems analysis:
Semi-structured interviews will be conducted. The interviews focus on the Egyptian mental health system and the identification of bottlenecks. To this end, a mix of key informants (e.g. scientists,

government and donor officials, health care workers or NGO workers) will be recruited to gain a range of perspectives on the mental health system in Egypt.

RCT:
This study applies a single-blind, randomised, two-arm, control group design. The N = 532 participants will be allocated to one of two conditions using a block randomization algorithm. Group 1 (SbS + TAU) has full access to the SbS self-help program with e-helper contact-on-demand and information on treatment-as-usual in Egypt. Group 2 (TAU) receives basic psychoeducation (based on the first session of SbS) and information on treatment-as usual in Egypt. Participants in both groups answer sets of questionnaires at the beginning of the study (T1), 6 weeks after starting the study (T2), 4.5 months after starting the study (T3) and 12 months after starting the study (T4). All assessments will be done online and are implemented in the SbS software. The participants will be aware of which group they were allocated to.

Step-by-Step:
SbS is brief (5 sessions) and has been designed by the WHO to primarily support people with symptoms of depression or general psychological distress. The intervention comprises of exercises to increase behavioural activation, including pleasurable activities and social support; and additional strategies to support this, including a stress management technique and positive self-talk exercises. SbS is available online (website) and offline (smartphone apps for iOS and Android) in Arabic and English. For additional support (i.e. contact-on-demand), participants can contact a team of trained non-specialist research assistants called “e-helpers”. The e-helper team is located in Berlin, Germany. It consists of Syrian Arabic-native speakers with a background in psychology or associated disciplines who reply to questions regarding the study, the SbS program or technical issues. E-helpers are trained and supervised by experienced mental health practitioners.

SbS follows a narrative approach. The story and its illustrations were adapted to the cultural context, considering linguistic and cultural nuances within the Syrian population. There are four versions of the SbS narratives, two male versions and two female versions. The text was adapted to appeal to a) people who do not have children and may be single and b) people who have children and are likely married. For each gender there will be two different styles of dress in the illustrations, reflecting local culture. The content of Step-by-Step was developed with experts in psychological care, e-mental health and global mental health. It has gone through extensive peer-review, with over 30 external experts reviewing the intervention.

Process evaluation and interviews on scaling up:
In-depth interviews will be conducted with a range of stakeholders to explore different views on SbS and how the program can potentially be scaled up. This includes a) Syrian refugees that have participated in the intervention, b) MHPSS providers and c) key informants in Egypt.

2.2 Previous experience with the methods

Internet-based psychological support-programs are effective in treating a variety of common psychological problems and have been successfully adapted for refugee populations. Prof. Christine Knaevelsrud at Freie Universität Berlin is one of the internationally leading research groups in the field of e-mental health and has extensive experience in the development, implementation and evaluation of internet-based therapeutic interventions. A main focus of previous research projects was to improve the access of populations with limited access to MHPSS (e.g. refugees, older patients, patients with special needs) to therapeutic care. The implementation of these projects was funded by the German

Research Foundation, by multiple Federal Ministries, as well as by the largest German health insurance company.

Prof. Roberts at the London School of Hygiene and Tropical Medicine is an expert on health care systems and policies. His team is a partner in the STRENGTHS project and responsible for the health systems analysis and interviews on scaling-up. The research group is part of several international research projects on health care in humanitarian crises.

The SbS program that will be used in this study in Egypt was recently tested in a feasibility trial with Syrian and Lebanese participants in Lebanon and is currently being tested in a pilot trial with Syrian participants in Germany. The project is supported by the original creators of SbS at the WHO.

2.3 Plan

Expected starting date

The study is estimated to start in June 2020.

Expected end date

The study is estimated to end in December 2021.

2.4 Timetable and relation of different study parts

The qualitative interviews for the health systems analysis will be conducted between June and December 2020. In parallel, the RCT will be conducted between June 2020 and December 2021. The process evaluation and interviews for scaling up will be conducted starting in December 2020 and will be completed by December 2021.

The health systems analysis and the RCT are independent study elements. However, the process evaluation is closely linked to the RCT because it aims at interviewing participants and staff. The interviews on scaling-up will refer to the results of the health systems analysis, e.g. in order to investigate the potential of SbS to fill in gaps or reduce bottlenecks.

2.4 Data collection

Nature of data collection

Health systems analysis, process evaluation and interviews on scaling-up:

Qualitative interviews will be conducted by trained interviewers using topic guides and last up to approximately 1 hour. All interviews will be conducted in person or by secure voice-over-IP (VoIP) / telephone. The topic guides for the health systems analysis cover questions regarding MHPSS services, access and coverage as well as quality and safety of MPHSS provision in Egypt. The topic guides for the process evaluation and scaling-up cover the user experience during the study, usability dimensions and potential ways of making SbS widely available.

RCT:

In the RCT, all assessments are conducted online, using electronic questionnaires that are accessed directly in the app.

Screening:

Before inclusion into the study, potential participants will be screened for increased psychological distress using the Kessler-10 Psychological Distress Scale (K10). Impaired functioning will be assessed using the WHODAS 2.0 (see below). Age and suicidality will be assessed using single-item screening questions.

Primary outcomes:
Psychological distress: Hopkins Symptom Checklist (HSCL-25)
The HSCL-25 consists of 25 items related to psychological distress. It includes subscales for depression (13 items) and anxiety (10 items). In addition, two items assess somatic symptoms.

Functioning: WHO Disability Assessment Schedule (WHODAS) 2.0
The WHODAS 2.0 is a generic assessment instrument assessing health and disability. It is used across all diseases, including mental neurological and substance use disorders.

Secondary outcomes:
PTSD symptoms: PTSD Checklist for DSM-5 (PCL-5)
The PTSD Checklist is a 20-item checklist corresponding with the 20 DSM-5 PTSD symptoms.

Self-defined problems: Psychological Outcome Profiles Instrument (PSYCHLOPS)
The PSYCHLOPS consists of four questions. It contains three domains: problems, function and wellbeing. The post-therapy version of PSYCHLOPS adds an overall evaluation question determining self-rated outcome ranging from “much better” to “much worse”.

Access to health services: Perceived access to health services will be measured through a questionnaire that was developed by the London School for Hygiene and Tropical Medicine (LSHTM) specifically for use in the STRENGTHS studies.

Cost of care: An adapted version of the Service Receipt Inventory (SRI) was developed for the collection of data on service utilization and related characteristics of people with mental disorders, as the basis for calculating the costs of care for mental health cost-effectiveness research. The questionnaire assesses the frequency of service use, so that service costs can be calculated.

User satisfaction: Client Satisfaction Questionnaire (CSQ-3)
The Client Satisfaction questionnaire is an easily scored and administered eight items measure that is designed to measure client satisfaction with mental health services. For reasons of parsimony, we will use the three items version, which uses the most salient items for the measurement of satisfaction with services.

Potential moderators or confounders:
Demographic variables: Demographic questions are part of the WHODAS 2.0 questionnaire. Questions on refugee status and flight from Syria were added.

Exposure to potentially traumatic events: Trauma list
Previous stressor exposure will be assessed using an adapted version of the Life Events Checklist. This is a widely used list of experienced or witnessed events, such as rape, serious injury, combat exposure, or the sudden death of a loved one.

Post-migration stressors: Post-Migration Living Difficulties Checklist (PMLDC)
The PMLDC examines the extent to which post-migration challenges had been of concern to the individual. We are using a 17-item adapted version of the scale that has consistently been identified as a predictor of mental health among displaced populations and has previously been used in Arabic speaking refugees.

Contamination of the control group:
To assess contamination of the TAU control group, a short set of questions assesses whether participants saw any of the SbS content or were taught any of the SbS skills.

Process variables:
The K10 questionnaire (see above) will be assessed weekly for 6 weeks. In addition, participants can choose to report on their momentary mood using a 5-item smiley-scale. Indicators of app usage will be tracked automatically (e.g. number of completed modules or number of completed exercises).

2.5 Power calculation

RCT:
The proposed sample size of N = 532 for the RCT is based on an estimated small-to-medium effect size in the SbS app group at 3-months follow-up. In a recent network meta-analysis on CBT delivery formats, unguided self-help had an effect size of 0.13 (95% CI: -0.13 to 0.39) against care as usual while guided self-help had an effect size of 0.52 (95% CI: 0.28 to 0.77) against care as usual. SbS fits between these two endpoints of the guidance continuum due to the contact-on-demand component that is prominently implemented in the app. Furthermore, studies with a direct comparison show that unguided interventions can be as effective as guided interventions in reducing clinical symptoms. This is the case if elements of the intervention (e.g. standardized texts) are designed to guide and motivate users throughout the intervention. The narrative approach of SbS was specifically designed for this purpose. Consequently, we estimated an effect of d = 0.4 for the power calculation.

Aiming at a power of 0.90 (alpha = 0.05, two-sided), we determined a required number of 133 participants per group. Considering the issue of higher dropout rates in internet- and smartphone-based interventions, we expect an attrition of 50% at 3-month follow-up. Consequently, we aim to include a total of N = 532 participants (n = 266 in the SbS + TAU group and n = 266 in the TAU only group).

Health system analysis, process evaluation and interviews on scaling-up:
The actual number of participants included in the qualitative assessments will be based on the principle of saturation. Depending on when saturation is reached, 5 to 10 key informants will be interviewed as part of the health systems analysis. As part of the process evaluation, 35 to 50 participants in the RCT will be interviewed. In addition, approximately 5 to 10 stakeholders will be interviewed on approaches to scaling-up. Here as well, recruitment will be stopped when saturation is reached.

2.6 Documentation of the assessments

Health system analysis and process evaluation:
If participants agree, the interviews will be audio recorded and transcribed. Alternatively, written interview protocols will be created. The audio files will be destroyed upon completion of the transcripts. Therefore, the health systems analysis will be documented in the form of written transcripts and interview protocols.

RCT:
All assessments are conducted in the form of electronic questionnaires within the SbS app. The data can be exported in the form of Comma-separated values (CSV) files.

2.7 Data Management

Qualitative interviews:

The transcripts and interview protocols will be pseudonymised by adding a code and removing any information which could identify the participant (e.g. names, email, phone number, job titles or places of work). Audio recordings will be deleted on completion of the transcripts. The code list will be stored physically separated from the data and the participant consent forms in a locked compartment at Freie Universität Berlin. Upon completion of the study, participants who chose to be informed about the results of the study will be contacted. Afterwards, the data will be anonymized by destroying the code list.

RCT:

All data will be handled electronically on the Step-by-Step platform, no hard copies of data files will be produced. Data will be downloaded on completion of the trial. On download, the data will be fully anonymized by replacing the username with a random code and removing all personal information. The data download can only be initialized by the study coordinator. It is protected by two-factor authentication (password + SMS security code) to prevent unauthorized or unintentional download.

The platform has been intensively tested, and a feasibility RCT has been conducted in Lebanon prior to the definitive RCTs, in order to test the randomization procedures and the data handling. The feasibility RCT showed that the data export is accurate.

Archiving:

Personal data will only be stored for as long as is necessary for the study to be carried out. Exported, anonymized data will be stored securely for 10 years on encrypted storage devices and will be shared within the STRENGTHS research consortium for cross-project analyses. Data on the SbS server (required for the online functionality of the app) will be deleted upon completion of the study. Due to the sensitive nature of the data, no data will be made available in public open data repositories.

3 Ethical considerations

3.1 Risks for participants

Adverse events:

Evidence from face-to-face treatments suggests that between 5% and 10% of all patients experience a deterioration of their symptoms during treatment. Other negative effects of face-to-face treatments have been discussed, such as lower self-esteem, self-accusation, and becoming dependent on the therapist. Little is known on negative effects of internet-based interventions when compared to face-to-face treatment. Like face-to-face treatments, possible negative effects include: a deterioration of symptoms, non-response, novel symptoms, drop-out, and severe adverse events (e.g. misuse of alcohol or drugs, deliberate self-harm, and suicidal ideation or attempts).

Stigmatization:

Participants may experience mental health related stigmatization if third parties learn of their participation in the study. There is also a risk of family members not agreeing with the use of a mental health program which could result in exerting pressure or domestic violence.

Unauthorized access to personal data:

The software can be used flexibly and at a place of choice, using a personal smartphone or a computer. If basic precautions for data protection are not observed (e.g. not choosing a secure password, no access control measures for devices or virus protection), there is a risk of unauthorized access to personal data.

Cost for mobile internet usage:
The app version of SbS will be downloadable via the Apple and Android app stores. Due to the large number of pictures and audio files, the initial download will be comparably large (approximately 200 MB). Depending on a user's smartphone contract and access to WiFi, downloading the app via a mobile connection can result in unexpected costs.

3.2 Potential gain for participants

Reduction of psychological distress:
SbS provides evidence-based intervention elements that were developed in close cooperation with mental health experts. Based on previous research, it is assumed that the use of SbS can reduce symptoms of psychological distress.

Improving access to care:
The use of SbS is less affected by typical barriers to accessing care such as regional unavailability of services, travel distance, costs, waiting lists or the language barrier. Furthermore, SbS provides a continuously updated overview of available mental health care and support services for refugees in Egypt. Therefore, the use of SbS could improve access to care.

3.3 Assessment of the risk-benefit ratio

Since the e-mental health intervention is non-pharmacological and there is a broad evidence base for its safe use, it is unlikely that adverse effects due to the intervention will occur during the study. For all the above-mentioned risks, risk management measures were implemented (see below). The scientific value of the project lies in its potential to gain a better understanding of the effectiveness of smartphone- and internet-based interventions for refugee populations. The treatment gap in this population is particularly large and effective digital interventions could be one approach to providing basic help at a large scale. Considering that the approach has been implemented safely in numerous other populations, it can be concluded that the potential benefits clearly outweigh the risks.

3.4 Risk management

Adverse events:
The intervention is based on evidence-based therapeutic techniques found to be safe for use in a range of populations. If potential symptom deterioration is detected in one of the weekly assessments, participants receive an in-app notification that encourages to review the list of available services and seek additional support.

Stigmatization:
The intervention seeks to ensure that it does not stigmatize participants in several ways: (1) The intervention is delivered through own devices that can be used in private, (2) participants will be given the choice as to how (email or message) they want to be contacted for reminders and whether they wish to receive notifications, (3) the intervention seeks to be non-pathologizing, it does not involve identifying diagnostic categories or use specific mental health terminology and (4) the intervention will also be provided as a web-version without the need to install an app that could be found by other users of a device.

Unauthorized access to personal data:
The app enforces complexity requirements for passwords during account creation. Access to administrative features such as the client list or the data download is protected by two-factor authentication.

The SbS app and all procedures involving the software were developed in compliance with the EU General Data Protection Regulation (GDPR), the German Data Protection Act (BDSG) and the Berlin

Data Protection Act (BlnDSG). The software was developed using state-of-the art security measures (e.g. SSL encrypted data transfer, code reviews and unit tests) to ensure participant data is protected.

Cost for mobile internet usage:
Users will be instructed to download the app through a non-mobile connection or to make sure that the download is covered. Data usage after the initial download will be kept to a minimum because no additional content will be downloaded and data transfer (e.g. of questionnaire data) will be very small in size.

3.5 Broader ethical issues that could result from the study
We don't anticipate any broader ethical problems, long-term disadvantages or other indirect effects on parties not directly involved in this research.

4. Participants

4.1 Participant selection
The SbS app was designed against the backdrop of the ongoing Syrian refugee crisis that led to the conclusion that local health care system in host countries were not sufficiently equipped to scale-up mental health care provision for Syrian refugees. Due to the large overall number of Syrian refugees in Egypt, the country was chosen as an implementation sites for a highly scalable digital intervention approach.

Health systems analysis:
In order to gain a deeper understanding of the local health care system, we will purposefully select a diverse range of respondents with expertise on topics related to mental health care provision in Egypt (e.g. mix of male/female, ages, roles and expertise). The participants will be contacted based on expert recommendations and public information (e.g. organization websites).

RCT:
The SbS app was co-developed with participation from Syrian refugees and is designed for use by Syrian refugees in Germany, Egypt and Sweden. Participants in Egypt will be recruited through outreach by Caritas, Alexandria in the Alexandria region. Contact-on-demand will be provided by a team of trained and supervised Syrians.

Process evaluation:
Completers and non-completers in the intervention group will be randomly selected and invited to participate in the process evaluation interview. In addition, all staff members will be interviewed. Stakeholders will be selected from the list of stakeholders created during the health systems analysis, through expert recommendations and public information (e.g. organization websites).

4.2 Number of participants
Health system analysis:
Depending on when saturation is reached, 5 to 10 key informants will be interviewed as part of the health systems analysis.

RCT:
We plan to recruit N = 532 participants. The participants will be randomized to:
A treatment condition (n = 266) in which participants have access to the SbS self-help program (including psychoeducation) with contact-on-demand and information on mental health services that are available in their country of residence (SbS + ETAU condition).

An enhanced treatment-as-usual control condition (n = 266) in which participants will only receive basic psychoeducation (based on the first session of SbS) and information on mental health services that are available in their country of residence (ETAU condition).

Process evaluation:
As part of the process evaluation, 35 to 50 participants in the RCT, approximately 8 staff members and approximately 5 to 10 stakeholders will be interviewed. Here as well, recruitment will be stopped when saturation is reached.

4.3 Inclusion criteria

Health system analysis:
In order to be eligible to participate in the health system analysis, a subject must be a key informant or stakeholder (e.g. NGO worker, psychologist, policy maker, researcher) with expertise on the Egyptian health care system or aspects thereof.

RCT:
In order to be eligible to participate in the RCT, a subject must meet all the following criteria:
1) Syrian displaced person (based on self-disclosure)
2) Arabic speaking with a basic level of literacy
3) Elevated levels of psychological distress (K10 > 15)
4) Reduced psychosocial functioning (WHODAS 2.0 > 16)
5) Access to an iOS or Android smartphone or a computer with internet connectivity

Process evaluation:
In order to be eligible to participate in the process evaluation, a subject must have participated in the study (completer or non-completer in the intervention group), must be a staff member (e-helper or case manager / supervisor) or must be a stakeholder (e.g. NGO worker, psychologist, policy maker) in the potential scaling-up of SbS in the Egyptian health care system.

4.4 Exclusion criteria

A potential participant who meets any of the following criteria will be excluded from participation in this study:
1) People who have plans to end their life (assessed with a screening question)
2) Minors (under the age of 18)

4.5 Relation between researchers and participants

The researchers stand in no professional or private relation to the participants.

4.6 Procedures to handle adverse events

Although likely uncommon, all adverse events (AEs) and serious adverse events (SAEs) reported spontaneously by the participant or identified through study measures at any time will be recorded by the e-helper, interviewer or research coordinator for immediate action and resolution where required. All AEs and SAEs will be reported to the STRENGTHS safety committee, an independent board chaired by Prof. Claudi Bockting (University of Amsterdam, Netherlands). The STRENGTHS Safety Board (SB) will monitor all ethical, legal and societal issues that arise within the STRENGTHS project. The SB will ensure that the trial and data collection are conducted in accordance with the International Conference on Harmonisation (ICH), the WHO Good Clinical Practice standards (GCP), Declaration of Helsinki and (inter)national laws such as the Medical Research Involving Human Subjects Act (WMO). The STRENGTHS SB will review any SAEs as soon as possible and any AEs each month. They will

determine any appropriate action in respect of ongoing study conduct. The safety, rights and wellbeing of the participants and research staff members will be reviewed, and interim analyses will be considered in case safety issues are (suspected to be) violated. Other issues that will be considered include privacy and intellectual property rights. Relevant issues will be discussed periodically (on a six-month basis) in a meeting, but if issues arise between these meetings, the SB will be requested to plan an additional meeting.

Suicidality during screening:
Prior to participation, applicants will go through the self-screening that also includes a screening question on serious thoughts or plans to end one's life. Suicidal participants will not be included in the study and will instead automatically receive information on where to find help as well as the following self-care tips from WHOs mhGAP-IG 2.0:

"You have answered that you have had serious thoughts about or may have a plan to end your life. We are concerned you have been feeling this way and suggest you speak to a professional at a health clinic as soon as possible.
If you think you are in immediate danger of harming yourself, please go directly to your closest health centre or emergency room. Or call your national emergency telephone number.
Talk to a trusted family member, friend or colleague about how you feel.
While such thoughts can be very intense, it is of utmost importance that you don't act on them. People in similar situations experience that professional help alleviates their pain, and that there are alternatives. We encourage you to reach out for help. Remember, you are not alone."

Participant safety after inclusion in the study:
If, during the study, an adverse event should occur (e.g. the participant discloses plans to end their life or there is a serious protection concern requiring assistance), e-helpers will follow manualized standard procedures and always inform the clinical supervisor who will guide the e-helper. These procedures include the following steps:

If risk is not imminent, the e-helper provides information on where to seek care and self-care tips (see above). Information on available services in Egypt will be regularly updated. The referral information comprises of a list of health care organizations, hospitals, Arabic speaking professionals and alternative sources of help (e.g. specialized phone hotlines).

If risk is imminent, the e-helper will:
[A] Ask participant for a contact phone number to provide support over the telephone as opposed to text. The participant can choose not to provide a phone number. In this case, the support will be continued using in-app messaging or email.

[B] Ask if participant can go see someone they trust and try to stay in touch with the participant until he/she has met a trusted person. Ask to speak with the trusted person.

[C] If [B] is not possible: Ask if participant can call someone trusted to come and see participant. Contact participant again to be sure, someone will come. Ask to speak with the trusted person.

[D] If [B] and [C] are not possible: Ask participant to provide 2 phone numbers of trusted persons. Explain, that participant will be called back in 5-10 minutes. Call trusted persons and request them to go see participant. Call back participant.

In addition to the procedures described above, e-helpers can reach out to Caritas, Alexandria. Local Caritas staff will then reach out to the participant by continuing to contact the participant via phone. If contact fails, Caritas will send staff to check the participants safety in person. Participants that require referral will be assisted in contacting and going to a local hospital with mhGAP-trained staff.

4.7 Will financial compensation be given?

Yes

4.7.1 Financial recompensation for study participants

Professionals (e.g. NGO workers, psychologists, policy makers) in the health systems analysis and process evaluation will not receive any financial compensation for their participation. Syrians who participate in the RCT will receive 150 EGP in cash or phone credit for their time after completing the 3-month follow-up assessment and again after completing the 12-month follow-up assessment. Syrians who agree to participate in a process evaluation interview receive 150 EGP in cash or phone credit for their time. Participants can choose whether they prefer cash or phone credit as compensation.

5. Information and consent

5.1 Are participants informed about the research and asked for consent?

Yes

5.1.1 How and at what stage will participants be informed and asked for consent?

Qualitative interviews:

Potential participants will be given an information sheet on the study when they are first approached. At the time of the interview, the information sheet will be provided again, and a consent form will need to be completed. Before the start of an interview, the interviewer will provide once again a short verbal explanation on the study and will answer any remaining questions the participant might have.
All participants will be asked to give written consent to participate. Verbal consent can be given if the individual's literacy is limited, provided that an impartial witness who is appointed by that individual signs the consent form on their behalf. Some interviews may be held by phone or VoIP and in these cases the information sheet and consent will be sought through e-mail confirmation. Verbal consent will also be sought prior to the telephone/VoIP interview.

RCT:

All research participants will be asked for individual, electronic informed consent. All participants will download the SbS app or go to the SbS website of their own accord and be provided with detailed information at the commencement of the intervention about what they can expect, and reminded that they are free to withdraw at any time and that non participation will not affect in any way their access to any other form of health care.
E-helpers will receive in-depth training on the informed consent process and will reply to questions. All data and informed consent will be collected online. Full information on the study will be provided in Arabic or alternatively English as part of the consent form before requesting consent. Respondents who decide to participate will be asked to electronically sign the consent form. To improve accessibility, the study information and consent form are audio supported.

5.2 Will children under the age of 18 be included?

No

5.3 Will persons with limited legal capacity be included?

No

6. Recognition of results

6.1 How is access to the research data guaranteed?

The study coordinator has unrestricted access to the research data via the SbS administration website. Only the coordinator account can access the CSV data export. The data download is protected by two-factor authentication and requires the coordinator password as well as an authentication code that will be sent to the coordinators phone number.
In the unlikely case that access through the admin panel is not possible (e.g. due to a technical issue), the coordinator can request a direct database export from the software developing company. This data download will only be provided to the coordinator in person.

6.2 Who is responsible for data processing, analysis and presentation?

The following persons are responsible for the data processing, analysis and presentation: Prof. Christine Knaevelsrud (Freie Universität Berlin), Prof. Bayard Roberts (London School of Hygiene and Tropical Medicine) and Sebastian Burchert (Freie Universität Berlin). These persons will have access to the raw data export files and the interview transcripts / protocols.

6.3 How and when will the results be published?

Results of this study will be published in peer-reviewed papers and presented at scientific conferences, e.g. the International or European Society of Research on Internet Interventions (ISRII / ESRII). The presentations will be given in 2020 and 2021. The papers will be published in 2021.

Furthermore, results will be made available to practitioners and policy makers who plan to implement Step-by-Step in their respective contexts. This communication will be done through the World Health Organization, who will disseminate Step-by-Step after completion of the RCTs in Germany, Sweden, Egypt and Lebanon.

6.4 How is participant privacy maintained in the context of the publications?

Publications will never contain personal information or data of individual participants. Data will always be presented in the form of statistical parameters that refer to the whole sample or large sub-groups. No individual scores will be made available, and the raw data set will not be published.

The only exception are quotes by individual participants that may be used in publications on the health systems analysis or the process evaluation. The quotes will always be fully anonymized (i.e. removing any information that could be linked to a specific person) and will be presented using only a broad description of the person's role (e.g. Syrian refugee, mental health care provider or policy maker).
