# Supplementary material for: Effects of a self-guided digital mental health self-help intervention for Syrian refugees in Egypt: A pragmatic randomized controlled trial
Source: PLoS Med. 2024 Sep 9;21(9):e1004460. doi: 10.1371/journal.pmed.1004460 (PMC11419380; doi:10.1371/journal.pmed.1004460)
Supplement: Attachment — Submitted filename: PLOS Peer Review 15.1.2024.docx [file pmed.1004460.s003.docx]

**Peer Review for "Effects of a Self-Guided Digital Mental Health Self-Help Intervention for Syrian Refugees in Egypt: A Pragmatic Randomized Controlled Trial"**

**Summary of the Research & overall impression:**

The study investigates the effectiveness of a self-guided digital mental health intervention, the WHO Step-by-Step programme, with optional contact-on-demand (COD) support among Syrian refugees in Egypt. The pragmatic randomised controlled trial involves 538 participants and evaluates outcomes such as psychological distress, functioning, PTSD symptoms, and self-identified problems. The findings indicate small but significant positive effects on psychological distress and functioning at the 3-month follow-up. Remission rates did not differ, and there were challenges with intervention dropout and low COD utilisation. Overall, the study offers valuable real-world insights into scalable mental health interventions for refugee populations and is a worthwhile study topic.

There are a number of comments that should be addressed to add clarity to the paper and to guide the reader along their journey, listed below.

| **Line number/ paper section** | **Remarks** |
| --- | --- |
| **Major Issues to be addressed:** | |
| Discussion stage  194-5 | **High Intervention Dropout:**  The study acknowledges a high intervention dropout rate. Investigating the reasons behind this is helpful for understanding the intervention's challenges and potential improvements. A discussion on participant engagement barriers should be included to add depth to this element of the paper.  Re: *“considering a drop-out of 50%”*  Why did you anticipate such a high dropout rate in advance? What informed this? What pre-emptive steps did you take to reduce this rate or to make the trial more inclusive and accessible? |
| Discussion stage  271 | **Low COD Utilisation:**  The study acknowledges a low usage of COD support. Investigating the reasons behind this is helpful for understanding this element of the intervention and potential challenges in its use, access etc.  What types of issues were communicated/messaged via COD? Did it cost users/require internet connectivity? Was it mainly technical issues or were users asking for connection to services or other health queries? Is accessing it free of charge? Do you require internet connectivity or data to use it?  Re: *“Participants could at any time reach out to e-helpers for help with*  *the intervention, app functionality, technical issues, or other topics, including referral to other services.”*  Was this available 24-7? And did they? Which services were sought after/recommended? How was the COD used, and when was it?  An analysis of the COD discussion/usages may shine a light on the usefulness of it (or not) and present potential lessons learned.  Can you explain further the profile of the Syrian COD contacts? Were they both male and female? Are they Syrian diaspora or also forcibly displaced? Did they have a similar background to the general app users? What understanding do they have of the Egyptian context? Of refugee life/challenges?  How was the COD marketed/promoted to users during onboarding? Upon reflection, do you think more could have been done to support users with COD or was its low uptake due to something else? |
| 196 & 203  203 & 261 | **Access/eligibility:**  A limitation of this study is that it is open only to literate, urban-located Syrian refugees. Can you explain the decision (limitation?) of this study to offer this app only to urban-based, literate refugees and not to rurally based refugees or those with literacy issues who might have less physical/traditional/face2face (or otherwise) access to a range of alternative services? Why were these decisions made?  And if, as stated on line 261, this app has audio features, which could make it more inclusive for the visually impaired or illiterate (and verbal consent is an option, as per the protocol), could you explain why you didn’t broaden your eligibility criteria also to include rural refugees or refugees with such disabilities or reading challenges? |
| Discussion stage | **Effect Size Reporting & Discussion:**  While the effects on psychological distress and functioning are statistically significant, the clinical significance remains a little unclear.  Addressing the practical implications of these small effects for the well-being of Syrian refugees in the Egyptian context would enhance the interpretation of the study's findings. To support the reader, it would be helpful to offer a basic comparison to understand the magnitude of the observed effects for those who are not familiar with the tools you used to measure findings. (e.g. do these results compare with general results from other ‘traditional’ (non-digital) interventions for improving mental health outcomes for Syrians who have experienced war and have been forcibly displaced?) This would help to place your findings in a larger picture of clinical/mental health practice. |
| Regarding the different tools you used to measure within this study (e.g. Kessler Psychological Distress Scale, WHODAS, HSCL-25, DSM-5 PTSD, (PSYCHLOPS, PMLD, etc.) | **Tool Strengths and Limitations:**  Were these different tools designed to measure stress, trauma, wellness, etc., in crisis-affected people? People from the MENA region? Or were they developed in the Western world for those in the western hemisphere? How applicable were these tools for the SbS users?  Were there limitations and challenges you found with the tools you applied? Did you have to edit or adapt them for Syrians, or for refugees, or the Egyptian context? Did you trial these tools with these groups before deciding to use them? What were the strengths and limitations of these tools for your study? |
| 464-467 | The protocol refers to a user satisfaction survey that investigates the participants as well as a 5-item smiley scale where participants can track their moods. Where can the reader find these results? Can you signpost this to the reader or include some of the highlights in this paper?  The protocol outlines that the study team will describe the existing MHPSS systems available for Syrian refugees in Egypt, whether they access these or not, and what the barriers are to accessing and receiving such care. Is this the same plan as the “health systems analysis interviews”? Where can the reader find these results? Can you signpost this to the reader or include some of the highlights in this paper? |
| As per the Protocol | **User-Centred Adaptations:**  The protocol outlines that the study team will identify ways to improve the programme and scale it up. Including preliminary insights or recommendations based on participant feedback would provide actionable guidance for refining the intervention in subsequent iterations. Could you highlight main findings here? I see there are remarks regarding further user-centred research to contextualise SbS, which are described as “posing major challenges” (line 517), but they are not outlined. What are they, and why do these cause such challenges?  The protocol outlines the plans for cost-effectiveness and cost-saving analysis. Where can the reader find these results? Can you signpost this to the reader or include some of the highlights in this paper? |
| 451 | **Syrian health delivery preferences and framing of health/wellness…:**  If the evidence is there to say that guided interventions prove to be more effective, what was the rationale for removing the guided element in your study?  Could you include some discussion regarding the preferences of Syrians in how they receive their health care (paternalistic versus patient-centred?) and how this could have tensions with a self-guided intervention versus a guided intervention with a health professional/e-helper? Are there barriers here for the user group in terms of their preferred health-seeking routes/methods?  It could enrich the discussion to reflect upon how different groups frame and understand health and wellness versus sickness and specifically mental health, which can be interpreted in a number of ways by different groups. What are the Syrian refugee health seeking behaviours and expectations? This could link to your comment regarding contextualisation or user-centeredness. Here is an interesting paper that discusses Arab constructs of suffering, health etc. and gives further support to illustrate this point and hopefully support you in this reflection. *“Utilization of Mobile Mental Health Services among Syrian Refugees and Other Vulnerable Arab Populations—A Systematic Review”* https://www.mdpi.com/1660-4601/17/4/1295 |
| Missing from paper | Can you include in the paper a comment regarding any contamination between the two trial groups, how this was managed, and what potential effects this may have/did have on the results? |
| **Minor Issues to be addressed:** | |
| 216 | Re: *“Participants received a compensation of 150 Egyptian pounds (EGP; equivalent to 9 USD) for the post and the follow-up assessments, respectively.”*  Could you address the potential bias associated with the participant incentives and your findings? What impact might this have had on your results? |
| 250 | Re: *“the intervention encompassed an introductory session and five brief content sessions”*  Could you explain what these five sessions include/cover? Does the app provide practical support in terms of referrals to local services or advice on human and refugee rights or supporting life in Egypt for new arrivals or similar? |
| 305 | Re: *“The use of CAU services was analysed descriptively and included in exploratory analyses to assess the role of additional help-seeking behavior”*  This could be very interesting to the reader. Can you also share this within the main article or as supplementary material? |
| 447 | Re: “The reason to develop a contact-on-demand approach was to maximize scalability.”  Can you explain a bit about why COD requires fewer personnel than the other e-helper model? Is COD available 24-7? Is COD considered scalable because it was not really used? If COD was more successful/had a higher usage rate, would it still be considered scalable/require less personnel? |
| 262 | Did you ask the users who/what else supports them outside of health facilities? |
| 114 | Re: *The responsiveness of local healthcare systems in host countries impacts long-term trajectories of mental health in refugee populations (7)”*  I read and interpret this as if the local healthcare systems are to blame for the mental health trajectories of refugees in Egypt. I do not think this is what you mean, and so I suggest updating this line to refer to something about the (lack of) capacities of local health systems to manage and support the mental health issues of refugees in Egypt for the long term. |
| 130 | Re: *“Given that over 60% of Syrians in Egypt are considered vulnerable and living below the poverty line, reaching this refugee population for targeted interventions is challenging.”*  Is it really their vulnerability/living beyond the poverty line the reason they are considered hard to reach? Can you elaborate on what exactly you mean here? I argue that poverty itself does not make them hard to reach; some very rich people are hard to reach. What is/are the root cause(s) of their hard-to-reach nature? (e.g. undocumented, living with host families rather than being found in a camp environment, etc…) |
| 180 | Re: *“received one short information session”*  What was the purpose of this session? Technical onboarding? Study information? Give a brief explanation and outline if both study arms received this or only one. And if only one study arm received this session, explain why you made this decision. |
| **Other Comments:** | |
| 277 | Could use the CAU acronym here. |
| 120 | Re: *“Egypt is a low and middle-income country (LMIC)”*  Is this a typo? I expected this to say “lower middle-income country” |
| 121 | Re: *“136.727”*    This format (using “.” rather than “,”) appears not to be standard English. Suggestion to standardise format to “136,727” |
| 179 | In line 179, I wonder what CAU consists of for Syrian refugees in the Egyptian context. I see it has been explained in lines 277-281. I would consider moving lines 277-281 to the end of line 179 to inform the rest of this section or at least mention to the reader that you will explain what CAU is in section “X”. |
| 182 | Re: *“randomly generated blocks of length 2 to 8”*  Clarify here what these numbers mean (e.g. cm, hours, SbS modules, duration of assessments). It is not clear to me. |
| 237 | Re: *“Additional measures included single-item demographic questions on gender, age, education, marital status, and occupational status.”*  I suggest referencing “Table 1 Demographic and baseline characteristics” at this line. |
| **Review Conclusion:** | |
| In conclusion, the study makes an interesting and welcome contribution to understanding the effectiveness of digital mental health interventions for refugee populations.  Congratulations to the study team for addressing these important and evolving issues.  Addressing the major and minor issues identified will strengthen the study's validity and provide valuable insights for both researchers and practitioners in the field of refugee mental health and for the future of digital health. | |
